# Effect of systemic inflammation on level of ferritin seminal in chronic renal male patient undergoing hemodialysis

**DOI:** 10.1186/1755-7682-7-23

**Published:** 2014-05-12

**Authors:** Gilmar Pereira Silva, Carlos Daniel De La Vega Elena, Fabiana Pirani Carneiro, Joel Paulo Russomano Veiga

**Affiliations:** 1 University Hospital of Brasilia, Street 12 North, Lot 3, Clear water, Brasilia, Federal District, Brazil; 2 Biochemical Immunohematology and transfusion Specialist of Hospital Italiano Garibaldi, Rosario, Argentina; 3 University of Brasilia, Brasilia, Federal District, Brazil; 4 Department of Nephrology, University Hospital of Brasilia, Brasilia, Federal District, Brazil

**Keywords:** Chronic renal disease, Ferritin, Semen, Sex hormones, Testis

## Abstract

**Background:**

Most hemodialysis patients present with chronic systemic inflammation characterized by the elevation of serum C-reactive protein (CRP) levels and/or the production of proinflammatory interleukins by the immune system in response to the hemodialysis process. Plasma ferritin(PF) is one of the parameters used to correct anemia. An PF level of >500 ng/mL is not recommended for correction of anemia because of the uncertainty of whether these levels are elevated because of anemia or a mere reaction to inflammation. we aimed to study the effects of inflammation on seminal ferritin (SF) levels and hypothesized that SF is not affected because of the testicular immune privilege.

**Methods:**

A prospective prevalence study was conducted at the Department of Hemodialysis of the University Hospital of Brasília (HuB) between June 2010 and July 2011. The sample included 60 chronic renal patients undergoing hemodialysis and 20 control subjects from the health promotion general outpatient clinic. All participants were males aged 18–60 years. Inflammation was assessed through serum CRP levels, and the testicular condition was determined by measuring sex hormone levels. In the patient group, inflammation was considered to be present when CRP was >5 mg/L (n = 27) and absent when CRP was ≤5 mg/L (n = 33). Control group (n = 20) CRP was ≤1 mg/L. Blood and semen were collected via arm venoclysis and after voluntary masturbation, respectively. CRP was measured by turbidimetry; PF, SF, and sex hormone levels by immunochemoluminescence. Statistical significance was set at p < 0.05.

**Results:**

There was no significant difference in mean SF levels among patients with inflammation (295.34 ± 145.39 ng/mL), those without inflammation (324.42 ± 145.51 mg/mL), and controls (335.70 ± 075.90 ng/mL; p = 0.49). There was no correlation between mean SF and PF levels in the patients with and without inflammation). All participants were eugonadal with mean serum FSH, LH, and testosterone levels of 3.76 ± 2.17 mUI/mL, 7.00 ± 3.53 mUI/mL, and 454.18 ± 173.08 ng/dL, respectively.

**Conclusion:**

Systemic inflammation did not significantly alter SF levels in eugonadal hemodialysis patients.

## Background

Ferritin (Fn) is an intracellular protein that stores iron in the atoxic form. It is present in most cells and is found in the extracellular environment as a result of cellular synthesis and secretion [1]. In clinical practice, plasma Ferritin (PF) is an important parameter for the correction of anemia, together with the transferrin saturation index and serum iron levels. Anemia is a very common clinical condition in chronic renal patients, particularly those undergoing hemodialysis, for several reasons [2].

## Methods

This prospective prevalence study was conducted at the Department of Hemodialysis of the University Hospital of Brasilia (HUB) between June 2010 and July 2011. A total of 60 voluntary chronic renal male patients undergoing three weekly sessions hemodialysis for >6 months and without receive iron intravenous there at least 15 days. 20 control subjects from the health promotion general outpatient clinic of the same hospital, had normal glomerular filtration rate (GFR), this is, over 90 mls/min/1.73 m^2^, without clinical comorbidities that interfere with iron metabolism were included. All subjects were aged between 18 and 60 years and were assessed for inflammation via serum CRP measurements and for testicular function via serum follicle stimulating hormone (FSH), luteinizing hormone (LH), and testosterone (T) measurements. The study was approved by the Research Ethics Committee of the Faculty of Medicine of the University of Brasilia under protocol 024/2009. In the patient group, inflammation was considered to be present when CRP was >5 mg/L (n = 27) and absent when CRP was ≤5 mg/L (n = 33) according to the recommendation of National Kidney Foundation [3]. In the control group, a normal CRP value (≤1 mg/L) was considered to indicate low cardiovascular risk [4].

The inclusion criteria were as follows: patients aged between 18 and 60 years, patients undergoing hemodialysis for >6 months, and the absence of acute or chronic liver disease, neoplasia and clinical/laboratory signs of acute or chronic infection/inflammation, positive serology for hepatitis B, C, and HIV, vascular access infection, leucocytosis, fever, hypoproteinemia, hypogonadism, infection of the urinary tract, orchitis and vasectomy.

Blood was collected before a hemodialysis session in the patient group and on a previously scheduled day in the control group, always between 8:00 a.m. and 10:00 a.m. in the clinical laboratory of the same hospital. Five milliliters of blood were collected by arm venoclysis without an anticoagulant. The blood was centrifuged at 3500 × *g* for 20 min, and the supernatant was collected in a centrifuge tube and maintained at −20°C until Fn, FSH, LH, T, and CRP measurements. On the same day of blood collection, seminal plasma was collected from the ejaculate after voluntary masturbation in the proper environment at 37°C. After 30 min of liquefaction, the resultant fluid was centrifuged at 3500 × *g* for 20 min, and the supernatant was collected in a centrifuge tube and maintained at −20°C for the measurement of SF levels. Serum CRP levels were measured by turbidimetry in the automatic analyzer BN II (Dade Berhing, kit Dade Berhing, USA). PF, SF, and sex hormone levels were measured by enzyme immunochemoluminescence using the automatic analyzer Immulite 2000/Siemens*.* Specific kits were used for quantification in addition to calibrators and controls recommended by the manufacturer. All statistical analyses were performed using SPSS® for Windows, version 20.0. After the analysis of sample distribution using the Shapiro–Wilk normality test, differences between three independent quantitative variables were evaluated using the Kruskal–Wallis test, while those between 2 independent quantitative variables were evaluated using the Mann–Whitney U test. Statistical significance was set at p < 0.05 to reject the null hypothesis.

## Results

The mean SF levels were comparable between patients and controls (311.33 vs 329.14 ng/L; p = 0.29; Mann–Whitney U test). Likewise, the mean SF levels according to the serum CRP levels were as follows: CRP > 5 mg/L (295.34 ± 145.39 ng/mL) vs CRP ≤ 5 mg/L (324.42 ± 145.51 ng/mL) vs CRP ≤ 1 mg/L (335.70 ± 075.90 ng/mL) as per the Kruskal–Wallis test (p = 0.49). There was no correlation between SF and PF levels in the patients with serum CRP > 5 mg/L and those with serum CRP ≤ 5 mg/L (p = 0.07 and p = 0.26, respectively, Pearson’s test).The sample under study comprised eugonadal patients with mean serum FSH, LH, and testosterone levels of 3.76 ± 2.17 mUI/mL, 7.00 ± 3.53 mUI/mL, and 454.18 ± 173.08 ng/dL, respectively, in the patient group.

## Discussion

SF is found in abundance in the seminal plasma, and Sertoli cells (SCs) produce approximately 70% of its contents [5].SCs have physiological functions that are primarily controlled by serum FSH levels and secondarily controlled by serum testosterone levels [6]. Therefore, the hormonal profile of the participants was investigated to rule out possible hypogonadism. The fact that the patients were eugonadal ensured glandular functional integrity and maintenance of the privileged testicular immune condition [7] and eliminated any bias caused by variations in SF levels as a result of decreased function of SCs due to changes in serum sex hormone levels, particularly FSH levels [8].

A few studies have investigated SF in humans and have focused mainly on its potential role as an antioxidant in populations with impaired fertility [9,10].

The effects of systemic inflammation on intratesticular Ferritin levels were investigated in an experimental study using an animal model in which Ferritin exhibited a behavior similar to that of PF, i.e., the intratesticular Ferritin levels increased in response to systemic inflammation induced by intraperitoneal injection of a lipopolysaccharide, thereby increasing the synthesis of intratesticular IL-6 [11].

To the best of our knowledge, this was the first study to investigate this effect in humans. Our results indicated that the mean SF values (Table 1) in the patient group (311.33 ng/mL) and control group (329.14 ng/mL) were approximately 3 times higher than those found in previous studies conducted on patients with impaired fertility, namely studies [10], 66.99 ng/mL and 101 ng/mL, studies [9]. This is probably because of the distinct reactivity of Ferritin to the various kits used, antibody types, other molecules that potentially interfere with the assays, and the ratio of L and H chains of Ferritin [8,12]. The most commonly used commercial kits measure basic isoferritin that is rich in L chains, such as PF [13], whereas measurement of acid or neutral isoferritin such as SF may be inadequate because of the lack of specific commercial kits [14].

**Table 1:** Seminal ferritin levels(ng/ml) in case and control

**Variable**		**Ferritin seminal (ng/mL)**				
		**n**	**Mean**	**Median**	**SD**^**1**^	**p-value**
Not considering inflammatory factor	Case	60	311.33	319.50	683.0	
	Control	20	329.14	346.00	467.0	0.29*
Secondlevels serum CRP	CRP > 5 mg/L	27	295.34	322.00	145.39	
	CRP ≤ 5 mg/L	33	324.42	317.00	145.51	
	CRP ≤ 1 mg/L	20	335.70	351.00	075.90	0.49**

^1^Standard Deviation; *Mann-Whitney Test; **Kruskal-Wallis test.

The difference in the reactivity of PF to various commercial kits has been reported by [15], who found a weak correlation among 6 distinct kits used in 43 healthy individuals. In another study, Ford et al. [16] found a difference of 150 ng/mL among the 6 kits used in 60 nurses with chronic renal disease.

Systemic inflammation, according to the definition (CRP > 5 mg/L), did not cause a significant change in the mean SF levels in the patients. Moreover, there was no significant difference in the mean levels between the patients and controls (Table 1). This result seems to be in line with our initial hypothesis. The testicular immune privilege is probably the main factor contributing to the stability of SF levels, and this complex and yet poorly understood mechanism may result from local tolerance and immune regulation mechanisms capable of controlling antigen-specific immune responses [17]. These mechanisms involve antigen sequestration by the hematotesticular barrier; secretion of immunosuppression factors, primarily by macrophages and SC, peritubular, and Leydig cells; limited number of T cells; and the presence of regulatory T cells responsible for the modulation of local and systemic immune responses [18,19].

Mechanisms of endocrine and paracrine (local) control lead to a fine control of Ferritin intratesticular levels, thus protecting germ cells from the toxic effects of excessive iron and ensuring optimal iron supplies to the various enzyme systems that produce the energy necessary for germ cell maturation [20].

On the other hand, the small sample used in our study may have contributed to the results. This sample was primarily a consequence of the inclusion criteria, particularly the frequent presence of chronic viral infection and participants’ fear of having their intimacy exposed during the study.

The lack of a linear correlation between SF and PF levels (Table 2) in the patient group is explained by the sensitivity of PF to the effects of systemic inflammation [21] and the stability of SF levels because of the testicular immune privilege.

**Table 2:** Correlations between the levels of seminal and plasma ferritins second levels serum CRP in the group case

**Group**	**n**	**Pearson’ test**	
		**r**	**p**
CRP < 5 mg/L	27	0.355	0.07
CRP ≤ 5 mg/L	33	0.200	0.26

Compared with the use of serum IL-6 levels for the same purpose, the use of serum CRP levels for the investigation and quantification of the inflammatory condition in the population under study may be questioned by some because of the intrinsic characteristics of the protein; however, it does not invalidate our results because other comparative studies have indicated a strong correlation between serum CRP and IL-6 levels during inflammation [22,23].

## Conclusion

Systemic inflammation does not cause significant changes in mean SF levels in eugonadal chronic renal patients undergoing hemodialysis, with the SF levels being similar to those observed in normal controls. To the best of our knowledge, this is the first report on this specific topic in relation to humans, and further investigations are needed to confirm the results.

## Abbreviations

CRP: Reactive-C protein; CS: Célua de Sertoli; Fn: Ferritin; FSH: Follicle stimulating hormone; HuB: University Hospital of Brasília; IL: Interleucin; LH: Luteinizing hormone; PF: Plasma ferritin; SF: Seminal ferritin; T: Testosterone; TNF: Tumor necrosis factor.

## Competing interests

The authors declare that they have no competing interests.

## Authors’ contributions

GPS drafted the manuscript. CDVE, FPC and JPRV critically reviewed it and makes addition. All authors declared the final version of manuscript.
